# DNA sequencing using polymerase substrate-binding kinetics

**DOI:** 10.1038/ncomms6936

**Published:** 2015-01-23

**Authors:** Michael John Robert Previte, Chunhong Zhou, Matthew Kellinger, Rigo Pantoja, Cheng-Yao Chen, Jin Shi, BeiBei Wang, Amirali Kia, Sergey Etchin, John Vieceli, Ali Nikoomanzar, Erin Bomati, Christian Gloeckner, Mostafa Ronaghi, Molly Min He

**Affiliations:** 1 Protein Engineering, Illumina Inc., 5200 Research Place, San Diego, California 92122, USA; 2 Engineering, Illumina Inc., 5200 Research Place, San Diego, California 92122, USA; 3 Bioinformatics, Illumina Inc., 5200 Research Place, San Diego, California 92122, USA

## Abstract

Next-generation sequencing (NGS) has transformed genomic research by decreasing the cost of sequencing. However, whole-genome sequencing is still costly and complex for diagnostics purposes. In the clinical space, targeted sequencing has the advantage of allowing researchers to focus on specific genes of interest. Routine clinical use of targeted NGS mandates inexpensive instruments, fast turnaround time and an integrated and robust workflow. Here we demonstrate a version of the Sequencing by Synthesis (SBS) chemistry that potentially can become a preferred targeted sequencing method in the clinical space. This sequencing chemistry uses natural nucleotides and is based on real-time recording of the differential polymerase/DNA-binding kinetics in the presence of correct or mismatch nucleotides. This ensemble SBS chemistry has been implemented on an existing Illumina sequencing platform with integrated cluster amplification. We discuss the advantages of this sequencing chemistry for targeted sequencing as well as its limitations for other applications.

## Abstract (web summary)

Next-generation sequencing technologies vary in performance, which is often measured by metrics such as sequencing speed, accuracy and read length. Here, the authors present a new sequencing by synthesis method that monitors polymerase binding to DNA, and suggest that this method has the potential to generate longer and faster reads.

## Graphical abstract

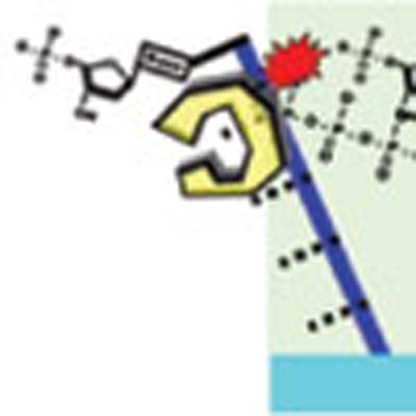

Next-generation sequencing (NGS) technologies have drastically reduced the cost of sequencing and placed a wide range of genomic analyses within the capabilities of many laboratories^1^,^2^,^3^,^4^. Multiple NGS technologies and platforms coexist and each offers unique advantages and disadvantages in sequencing metrics including speed, accuracy, read length, scalability, costs of instrument and reagents and simplicity of workflow^2^,^5^. One of the most important emerging applications of NGS is clinical sequencing, which promises to have a profound impact to human health. For clinical sequencing, the most important metrics are cost, accuracy, fast turnaround time (TAT) and integrated sample to answer workflow. Whole-genome sequencing is still relatively expensive, slow and the data are too complex to interpret for routine clinical applications^6^. Therefore, targeted sequencing that allows researchers and clinicians to focus on specific gene panels is most widely used because of its low cost and easier data interpretation.

The most commonly used targeted sequencing methods are based on Sequencing by Synthesis (SBS), including those from Ion Torrent and Illumina. Ion Torrent’s SBS method detects the byproduct of natural nucleotide incorporation, H^+^, by ISFET sensors^7^. The usage of natural nucleotides results in several advantages including lower cost and longer reads. Using semiconductor sensors for detection can make the platform highly scalable and cost effective. However, to be widely used for targeted sequencing in the clinical space, there are two outstanding concerns with Ion Torrent technology. First, DNA amplification using emulsion PCR in oil droplets is cumbersome and difficult to automate and integrate. Second, the accuracy of homopolymer-calling needs to be improved.

Illumina’s SBS offers two distinct advantages over the other SBS platforms: high accuracy and an integrated and automated workflow. Both of the attributes are critical to clinical sequencing. Since all the nucleotides are incorporated one at a time, homopolymer estimation is not required, which garners an advantage in sequencing accuracy^8^,^9^. However, the cyclical incorporation and deblocking of unnatural nucleotides in an ensemble population leaves room for Illumina to improve TAT and read length.

Prior studies have indicated that it might be possible to use polymerase kinetics for sequencing, but no actual DNA sequencing has been demonstrated^10^,^11^,^12^. The combination of co-crystal structures of DNA polymerase in action^13^ and fast mixing approaches, such as rapid-quench flow and stopped-flow^14^,^15^, has provided unprecedented kinetics and mechanistic insights into polymerase function^16^. These studies have shown that in the presence of a correct nucleotide, DNA polymerase forms a stable ‘closed’ ternary complex with a DNA template and the nucleotide, leading to nucleotide incorporation^17^. In the presence of a mismatch nucleotide, a transient and ‘ajar’ ternary complex is observed, which favours the fast dissociation of the polymerase from the DNA template^18^. As a result, it can be inferred from these observations that the binding kinetics of polymerase to DNA template in the presence of correct and mismatch nucleotides can be discriminated and subsequently used for DNA sequencing.

Using a labelled polymerase as the signal readout, we demonstrate the proof-of-concept of an ensemble SBS sequencing chemistry that monitors polymerase/DNA-binding kinetics (SPDBK) in real time for base discrimination. We present the theory and bulk kinetics results followed by DNA sequencing data for a single DNA template as well as a ΦX174 genome on an existing Illumina platform. This sequencing chemistry has unique advantages for targeted sequencing by combining the best of Ion Torrent and Illumina SBS chemistries. First, the use of natural nucleotides yields faster and longer reads, and second, the use of Illumina’s on board cluster amplification enables an integrated and fully automated workflow. We discuss the potential of this hybrid technology for clinical sequencing, as well its limitations for other genomic applications.

## Results

### Theory of binding kinetics for base discrimination

**Figure 1:**
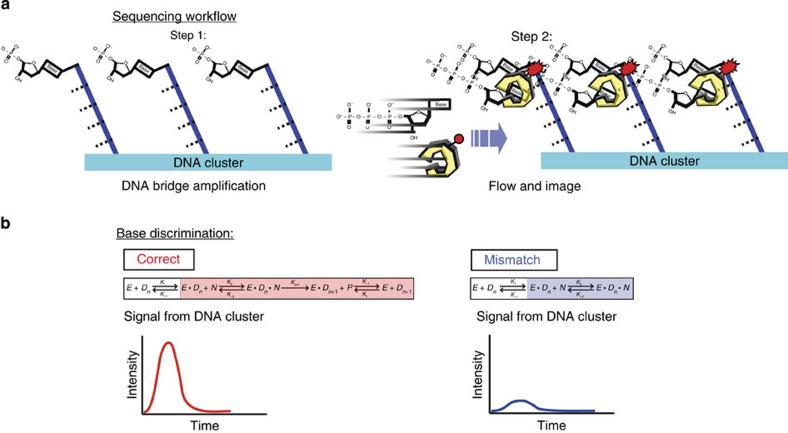
SPDBK workflow and base discrimination. (**a**) The sequencing workflow for this sequencing chemistry. In Step 1, the DNA is clustered in a flow cell. In Step 2, nucleotides are introduced into the GA flow cell in a cyclic fashion (**a**) and the polymerase/DNA-binding kinetics is imaged in real time (**b**). (**b**) Base discrimination is determined via polymerase/DNA-binding kinetics. Through the synchronization of polymerase/DNA binding by rapid mixing, the addition of the correct nucleotide gives rise to the more stable tertiary signal complex (SC) formation and subsequent catalytic chemistry step, *k*_pol_. The duration of this event is dictated by both the catalytic step and the nucleotide binding to the active site. As a result, the concerted binding of polymerase to DNA and the correct nucleotide gives rise to a pre-steady-state peak, which eventually turns over to the incorrect state and returns to steady-state equilibrium binding (Correct). The kinetics of the mismatch event is primarily dictated by *k*_−1_ or the enzyme dissociation from the template DNA and hence, the signal complex is less stable and there is little synchronized enzyme/DNA binding (Mismatch).

We monitor the binding of labelled polymerase to DNA in bulk solution and on immobilized DNA clusters^1^ by detecting the emission signal from the labelled polymerase/DNA complex formation in a total internal reflection fluorescence field (Fig. 1). For the concept of sequencing using polymerase/DNA-binding kinetics (SPDBK), we propose the following simplified kinetic model.


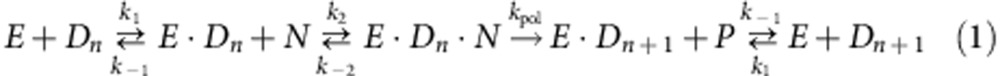


This model has been widely accepted by the polymerase research community to describe the minimal model for polymerase/DNA and nucleotide binding, nucleotide incorporation and DNA dissociation. The first step in the kinetic pathway described in equation (1) is the reversible binding of the polymerase to the DNA, *k*_1_, which is concerted or followed by the nucleotide substrate binding to the enzyme/DNA complex to form a tertiary complex, *k*_2_. Following the binding of the correct nucleotide in the polymerase active site, the enzyme undergoes a conformational change to the closed conformation (kinetically collapsed into *k*_2_) that commits the enzyme to forward catalysis (*k*_pol_). The simplification of nucleotide ground state binding and substrate induced fit has led to the use of *K*_d,apparent_ (*K*_d,app_) to describe one step nucleotide binding (*k*_2_) in the minimal model.

Since these reactions are run well above the *K*_d_ for the correct nucleotide binding to the polymerase active site and at high ionic strength, base discrimination is primarily driven by both enzyme/DNA and enzyme/DNA/dNTP-binding kinetics. Translocation, a conformational change back to the ‘open’ state, and *PP*_*i*_ release occur following nucleotide incorporation, and can collectively be grouped as post-chemistry steps. The specific order of post-chemistry steps is yet to be elucidated. With the minimal model described by equation (1); however, the steps following chemistry are assumed to be kinetically fast and can be omitted. Depending on the processivity of the polymerase, the enzyme can bind the next correct nucleotide or dissociate from the DNA following the post-chemistry steps (*k*_1_). In the proposed sequencing scheme, the polymerase is labelled and the recorded signal arises from any polymerase/DNA complex shown in equation (1).

It is possible to express the rates of consumption and formation of these complexes mathematically in the presence of a nucleotide:


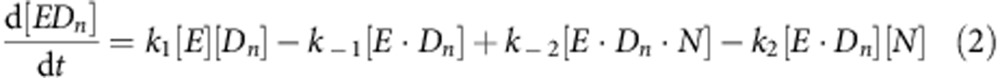



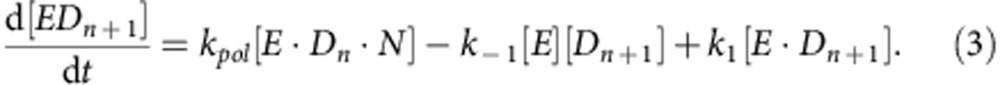



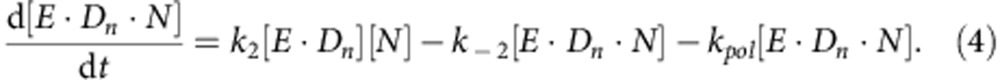


Since it is not possible to distinguish a unique signal to differentiate between *E*·*D*_*n*_, *E*·*D*_*n*+1_, and *E*·*D*_*n*_·*N* complexes, we redefine these terms as the signal complex, SC,


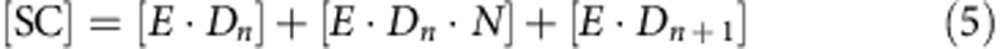


Combining the equations (2)–(5), 3, 4, 5, we can simplify the definitions for the rates of [SC] formation in the presence of correct and mismatch nucleotides:


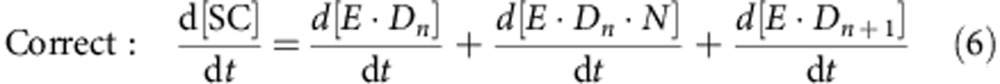



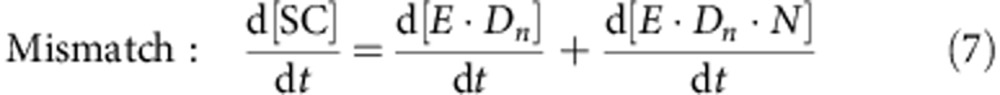


It is observed that when polymerase is mixed with mismatch nucleotides, the binding activity reaches steady state, governed by equation:


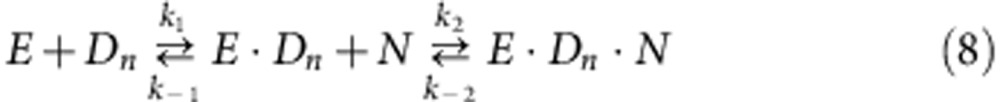


In the presence of a correct nucleotide, however, the additional chemistry step is involved before the steady state is reached (equation (8)).

**Figure 2:**
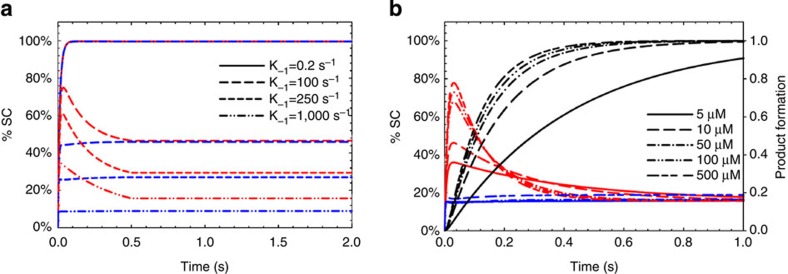
Theory of binding kinetics for base discrimination. (**a**) Simulation of DNA off-rates (k_-1_) dependence of SC formation in the presence of correct (red) and mismatch (blue) nucleotides. Curves represent the time-dependent formation of signal complex (SC) that includes *E*·*D*, *E*·*D*·*N*_corr_ and *E*·*D*_*n*+1_ (for the correct nucleotide) or *E*·*D*, *E*·*D*·*N*_mismatch_, and *E*·*D*_*n*+1,mismatch_ (for the mismatch nucleotide). % SC is defined as the percentage of total enzyme that forms the signal complex (SC). Under low ionic condition (solid traces), which was simulated as a very slow DNA off-rate, *k*_−1_=0.2 s^−1^, the SC is relatively stable. The % SC complex in the presence of the correct (red curve) and mismatch (blue curve) nucleotides quickly reaches 100% and subsequent steady-state equilibrium. In contrast, at very high ionic conditions (dashed traces), which was simulated as very fast DNA off-rates, *k*_−1_≥100 s^−1^, SC is much less stable. The disparity of rate and % SC formation with correct versus mismatch nucleotide is observed and can be used as a basis for base calling. (**b**) Simulation of correct (red) and mismatch (blue) nucleotide concentration dependence on SC formation. Simulation was performed under conditions such that the ionic strength of the buffer resulted in a DNA off-rate (k_−1_) of 500 s^−1^. Increasing the nucleotide concentration from 5 μM (solid trace) to 500 μM (dashed traces) resulted in an increased % SC from 0.4 to 0.8. On the contrary, the % SC remained at 0.2 when mismatch nucleotide increased from 5 μM (solid traces) to 500 μM (dashed traces). The altered kinetics of correct nucleotide tertiary complex due to increased ionic strength resulted in complete product formation with a k_pol_ of 9 s^−1^ and a K_d,apparent_ of 30 μM. The rate of product formation (black traces) is also shown. Simulations were performed using a KinTek Global Kinetic Explorer (KinTek Corp. Austin, TX).

In the simulation experiments, we model the change in signal to be directly proportional to the concentration of SC. At lower ionic strength, the affinity of the enzyme for DNA is high, which is expressed as a slow DNA off-rate (*k*_−1_). When the low ionic strength condition is simulated (*k*_−1_=0.2 s^−1^), the results revealed that the equilibrium favours a stable binary complex and the time-dependent percent SC formation (% SC) for correct (red curve) and mismatch (blue curve) nucleotides are similar (Fig. 2a). However, at higher ionic strength or increased DNA off-rate (*k*_−1_), the affinity of the enzyme for DNA decreases and the equilibrium favours minimal binary complex formation^19^,^20^. For simulated conditions with *k*_−1_>100 s^−1^, the correct nucleotide binding becomes increasingly important to stabilize the SC. As a result, we observed significant differences in the rate and percent SC formation for correct versus mismatch nucleotide, which forms the basis for base calling. We can further improve base discrimination by increasing the nucleotide concentrations, as revealed in the simulation results (Fig. 2b). By combining high ionic strength and nucleotide concentration, we show that for >100 μM nucleotide and >250 s^−1^ off-rate, the maximum % SC for matched nucleotide and mismatched nucleotide are ~80 and 17%, respectively. This ratio predicts that our discrimination of match versus mismatch bases should be greater than fivefold.

### SPDBK base discrimination in ensemble solution

While it was possible to theoretically predict the feasibility of base discrimination using a polymerase/DNA-binding kinetics model, the mixing of the reacting species is assumed to be instantaneous in the simulation. This assumption insures that all polymerase/DNA-binding events are perfectly synchronized. If polymerase/DNA-binding events are asynchronized, the SC formation is dispersed over time, which leads to a flattened signal response, and subsequently resembles a mismatch event. We minimized contributions from diffusion constants and mixing times on SC complex formation for the kinetics model shown (1) by using rapid reagent delivery and mixing systems, such as stopped flow and quench flow techniques^14^,^15^.

**Figure 3:**
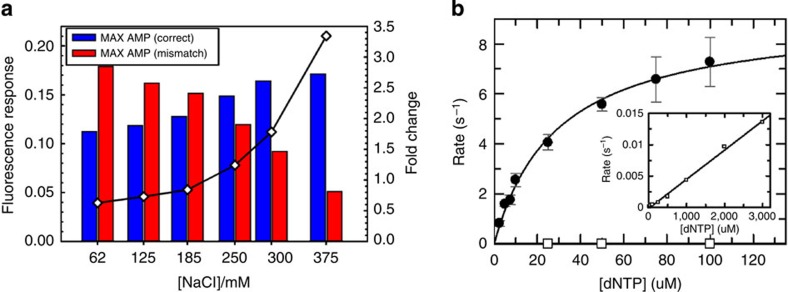
SPDBK base discrimination in ensemble solution. (**a**) Stopped-flow nucleotide-induced fluorescence response shows NaCl concentration dependence. Correct (dCTP) and mismatch (dATP) nucleotides were rapidly mixed with BSU polymerase- and FAM-labelled 19/36mer in the presence of various NaCl concentrations. An increase in NaCl resulted in an increased fluorescence response for the correct nucleotide (solid) and decreased response for the mismatch (hatched). The net result is a NaCl-dependent 3.5-fold increase (◊) in correct signal versus mismatch from 62.5 to 375 mM NaCl. (**b**) Quench flow nucleotide concentration dependence of product formation under high salt conditions. Increasing concentrations of correct (dCTP) or mismatch (dATP) nucleotides were rapidly mixed with BSU polymerase and 19/36mer in 300 mM NaCl buffer. The resulting time dependence of product formation for each nucleotide concentration was fit to a single-exponential equation to obtain a fitted rate with error (Supplementary Fig. 1c,d). The nucleotide concentration dependence of the obtained rates for correct ([cirf ]) and mismatch (□) product formation were fit to a hyperbolic equation to derive values of 9.15±0.4 s^−1^ and 29.1±2.9 μM for *k*_pol,dCTP_ and *K*_d,app,dCTP_, respectively. The specificity constant (*k*_pol,dCTP_/*K*_d,app,dCTP_=*k*_cat_/*K*_m_) for the correct nucleotide is 3.1±0.1 × 10^−1^. The nucleotide concentration dependence on the rate of mismatch incorporation could not be saturated due to experimental limitations on nucleotide concentrations. The resulting nucleotide concentration dependence of the observed rates were fit to a linear equation with a slope (corresponding to the specificity constant) of 4.6±0.1 × 10^−6^ μM^−1^ s^−1^. The specificity constants for correct and mismatched nucleotides were used to calculate a discrimination value of 6.6±0.3 × 10^4^ in high NaCl conditions.

From the simulation results, it is clear that a large polymerase/DNA off-rate *k*_−1_ and high nucleotide concentrations are critical parameters for base discrimination. Larger DNA off-rates, *k*_−1_, were achieved by increasing the salt concentrations of the reaction buffer. As was revealed in the simulation experiments, the correct nucleotide binding becomes increasingly important to stabilize the SC (Fig. 2b). Thus, when DNA-binding affinity of the polymerase was decreased at high ionic strength, nucleotide concentrations were increased to drive the reaction forward in the presence of the correct nucleotide. For bulk solution measurements, we determined the formation and consumption of SC complex by monitoring the binding kinetics of polymerase to dye-labelled DNA by fluorescence-quenching responses, which arise due to environmental changes surrounding the dye on polymerase binding^21^. The stopped flow fluorescence response traces were normalized and the maximum fluorescence-quenching responses were determined and correlated with the extent of synchronized polymerase/DNA binding (Supplementary Fig. 1a,b). As NaCl concentration was increased and the nucleotide concentration was held constant, we observed an increased fluorescence change for the correct nucleotide (blue), while a decrease for the mismatch (red, Fig. 3a). The 3.5-fold increase in correct signal versus mismatch at 375 mM NaCl demonstrated that we can achieve base discrimination experimentally by increasing DNA off-rate, *k*_−1_, which is consistent with the simulation results (Fig. 2a).

We also explored nucleotide concentration effects on product formation under a high salt condition using quench flow measurements (Fig. 3b). We used the specificity constants for correct and mismatched nucleotides to calculate a discrimination value of 6.6±0.3 × 10^4^ μM^−1^ s^−1^ in high NaCl conditions. In addition to providing an estimated apparent *K*_d,app,dCTP_ for the nucleotides under higher ionic strength, these measurements also demonstrate that high mismatch discrimination under these conditions is maintained, which is critical for the accuracy of a sequencing technology.

### SPDBK base discrimination in DNA clusters

As was previously mentioned, the extent of signal above background can be influenced by the speed of reagent delivery and mixing. In the stopped flow sample chamber, the mixing times are on the order of milliseconds and mixing occurs in a three-dimensional volume. For sequencing applications, the DNA templates are immobilized on a surface and a microfluidic flow cell is used. In this configuration, reagent delivery and mixing are slower and less efficient and may result in some asynchronization of polymerase/DNA-binding kinetics. To address these potential limitations, we delivered reagents to the flow cell using a syringe pump running at 4 ml min^−1^ and imaging was always performed in proximity to the inlet of the flow cell to minimize flow impedance effects that arise farther downstream in the flow cell. With immobilized DNA templates, we directly record the time evolution of the SC on the DNA clusters for correct and mismatch nucleotides.

**Figure 4:**
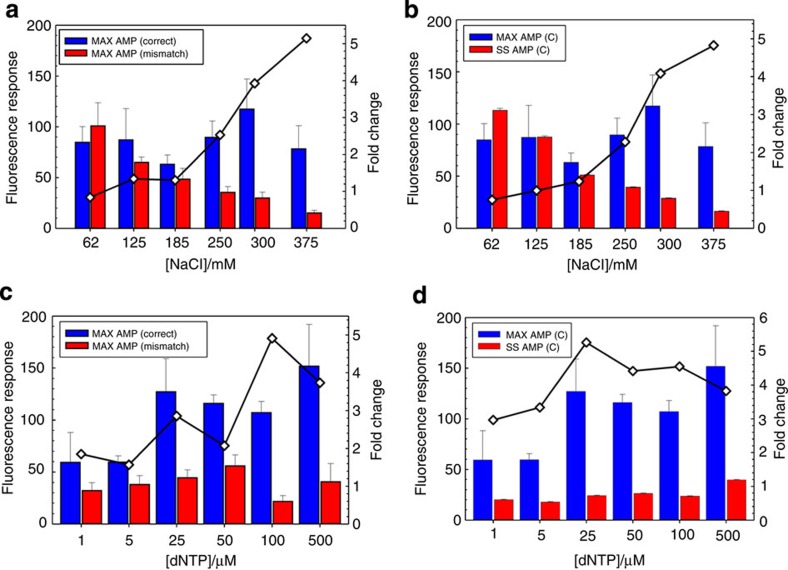
SPDBK base discrimination in DNA clusters. The SS AMP is defined as the point at which the fluorescence response returns to a baseline. For each [NaCl] and [dNTP] data point, the averages of the maximum amplitude (MAX AMP) and steady-state amplitude, correct (SS AMP (C)) are calculated from the three independent correct and mismatch measurements following the synchronization step. The error bars represent the s.d. for each of the conditions calculated from three independent measurements that are recorded on the same clusters at different positions on the calibration template. Since each condition is run on the same flow cell in the same lane, the error bars on the measurement are mostly reflective of differences in fluidic delivery, and potential signal loss due to incomplete extension, misincorporation, or read forward. (**a**) The peak amplitude correct (red bars) and mismatch nucleotide responses (blue bars) were determined from the raw traces (Supplementary Fig. 3a,b) over a range of NaCl concentrations, 62–375 mM. The maximum fluorescence response from the correct and mismatch traces are ascribed to the extent of synchronized enzyme–DNA binding. The net result was a NaCl-dependent fivefold increase (◊) in correct signal versus mismatch from 62.5 to 375 mM NaCl (**b**) For higher NaCl concentrations, the MAX AMP (blue) increased, while SS AMP amplitudes (red) decreased. The ratio of the MAX AMP/SS AMP increased approximately fivefold at the highest NaCl concentrations. (**c**) To find the ideal dNTP concentrations for maximum discrimination power, a range of dNTP concentrations of 5–500 μM were introduced into flow cells with 150 nM labelled BSU polymerase at a fixed NaCl concentration (300 mM). The peak amplitudes of correct (red bars) and mismatch nucleotide responses (blue bars) were determined from the raw traces (Supplementary Fig. 3c,d) over a range of dNTP concentrations from 1 to 500 μM. The net result was a dNTP dependent 5-fold increase (◊) in correct signal versus mismatch at 100 μM. (**d**) The MAX AMP (blue bars) response peaked around 100 μM while the SS AMP (red bars) increase slightly to yield a fourfold improvement in discrimination at a nucleotide concentration around 50 μM.

Background corrected time traces were extracted for the individual clusters as described in materials and methods (Supplementary Fig. 2) from a single region of interest (ROI) of the field of view (FOV). Approximately 500 time traces from the randomly selected ROI were averaged (Supplementary Fig. 3), and the maximum amplitudes (MAX AMP) and steady-state amplitudes (SS AMP) were determined for each experimental condition. Correct or mismatch nucleotides, at a fixed nucleotide concentration of 100 μM, were introduced into the flow cells with 150 nM labelled BSU polymerase over a range of NaCl concentrations of 62–375 mM. The nucleotide concentration of 100 μM was selected based on the quench flow results for correct dCTP (*K*_d,app,dCTP_=29.1±2.9 μM). Similar to the stopped flow data results, as we increased the NaCl concentration and held the nucleotide concentration constant (Supplementary Fig. 3a,b), we observed that the maximum amplitude (MAX AMP) of the fluorescence response for the correct nucleotide (blue bars) was increased, while the response for the mismatch (red bars) decreased slightly (Fig. 4a). We calculated a net fivefold increase in correct signal versus mismatch when NaCl concentration was increased from 62.5 mM to 375 mM. At low ionic strength, the decreased DNA off-rates (small *k*_−1_) incurred higher background on the flow cell substrates, which created a decreased fluorescence response above background for both correct and mismatch nucleotide binding (Supplementary Fig. 3a,b). As the DNA off-rates were increased at higher ionic strength (larger *k*_−1_), we measured fluorescence responses above background and improved discrimination of correct versus mismatch nucleotide. The steady-state amplitude (SS AMP) is correlated with the reaction completion and the stability of the SC. In addition to MAX AMP, the ratio of MAX AMP/SS AMP provided a second discrimination metric, which can be implemented for homopolymer calling (Supplementary Fig. 5). As the NaCl concentration increased from 62 mM to 375 mM, we observed that the ratio of MAX AMP/SS AMP increased by more than fivefold (Fig. 4b).

In a separate assay, we examined the nucleotide concentration dependence of base discrimination (Supplementary Fig. 3c,d). At elevated nucleotide concentrations, the MAX AMP of the correct nucleotide (blue bars) versus the mismatch response (red bars) increased until the nucleotide concentration was titrated above 100 μM (Fig. 4c). At 100 μM, we calculated a fivefold increase for the correct signal versus mismatch. At 500 μM, we measured a drop in the overall discrimination. We attribute this to an increase in steady-state amplitude, likely resulting from increased complex formation driven by high concentration of the nucleotides. At the same time, we observed a fivefold improvement in the MAX AMP/SS AMP ratio with increasing correct nucleotide concentration from 1 to 100 μM (Fig. 4d). From these preliminary discrimination results, we concluded that it is feasible to perform correct versus mismatch base discrimination with immobilized DNA clusters in a flow cell using polymerase/DNA-binding kinetics. These results also provided initial conditions to test the practicality of using this chemistry for DNA sequencing.

### SPBDK of synthetic monotemplate

**Figure 5:**
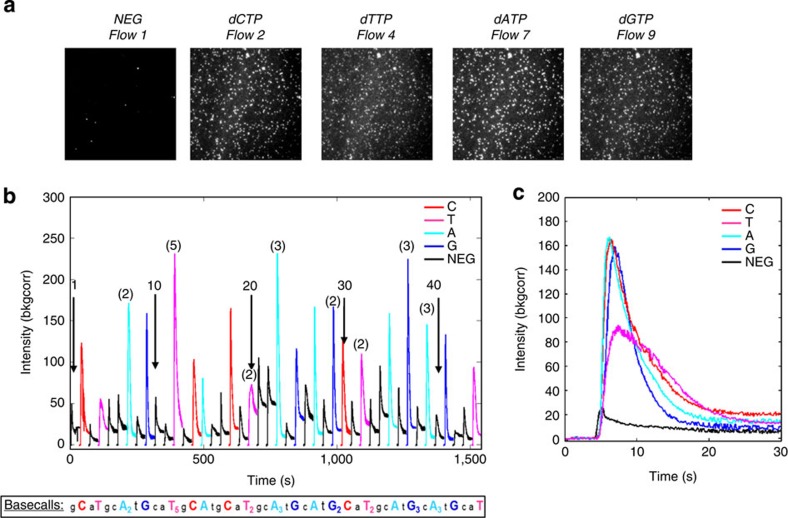
SPBDK of synthetic monotemplate. (**a**) Raw images averaged from selected individual flows. Example images for each of the nucleotides from indicated flow numbers were averaged from the time lapsed frame numbers, 50 to 150, which corresponds to 10 s of data. These frame numbers capture the rise, peak and fall of sequencing chemistry. (**b**) From a ROI in the FOV for a synthetic template, ~357 time traces were extracted and background corrected from each DNA cluster and for each individual flow. The 357 time traces were averaged and colour coded to indicate the correct sequence. Numbers are given as reference points for indicating flow numbers and numbers in parentheses indicate expected homopolymer repeats for the given flow. Flow order was arbitrarily chosen as ‘G’, ‘C’, ‘A’ and ‘T’. Correct base calls are indicated in capitalized coloured text and homopolymer repeats are indicated by subscripts, and negative flows are indicated by lower case text. (**c**) Averaged time traces for flows 3, 9, 18, 27 and 44, which correspond to negative, G, C, A and T 1-mer flows, respectively.

Flow cells were clustered with a single synthetic DNA template, whereby the first correct incorporation base was dCTP. We introduced reaction buffers that contained labelled BSU polymerase and one of the four nucleotides into the GA flow cell in a sequential automated fashion at a flow rate of about 4 ml min^−1^. In these experiments, we introduced 44 flows in a sequential flow order ‘G’, ‘C’, ‘A’ and ‘T’. The imaging set-up and settings were identical to those used in the salt and nucleotide titration experiments. From a randomly chosen ROI from the FOV, individual correct G, C, A, T and mismatch flows time lapsed images were time averaged from frames 50–100 (Fig. 5a). While we observed dark images from the mismatch nucleotide flows, the correct nucleotide flows yielded significantly brighter images. The time traces averaged from 357 DNA clusters in a random ROI clearly showed high intensity peaks for correct nucleotides and low signal for mismatch nucleotides (Fig. 5b), providing proof that base calling using polymerase/DNA-binding kinetics is indeed feasible. Interestingly, we also observed differential intensity levels for homopolymers, indicating the possibility for homopolymer discrimination. In addition, we can extract the chemistry reaction time of 15 s per nucleotide from the averaged flows for each of the individual bases (Fig. 5c).

### Homopolymer and base calling in SPDBK

**Figure 6:**
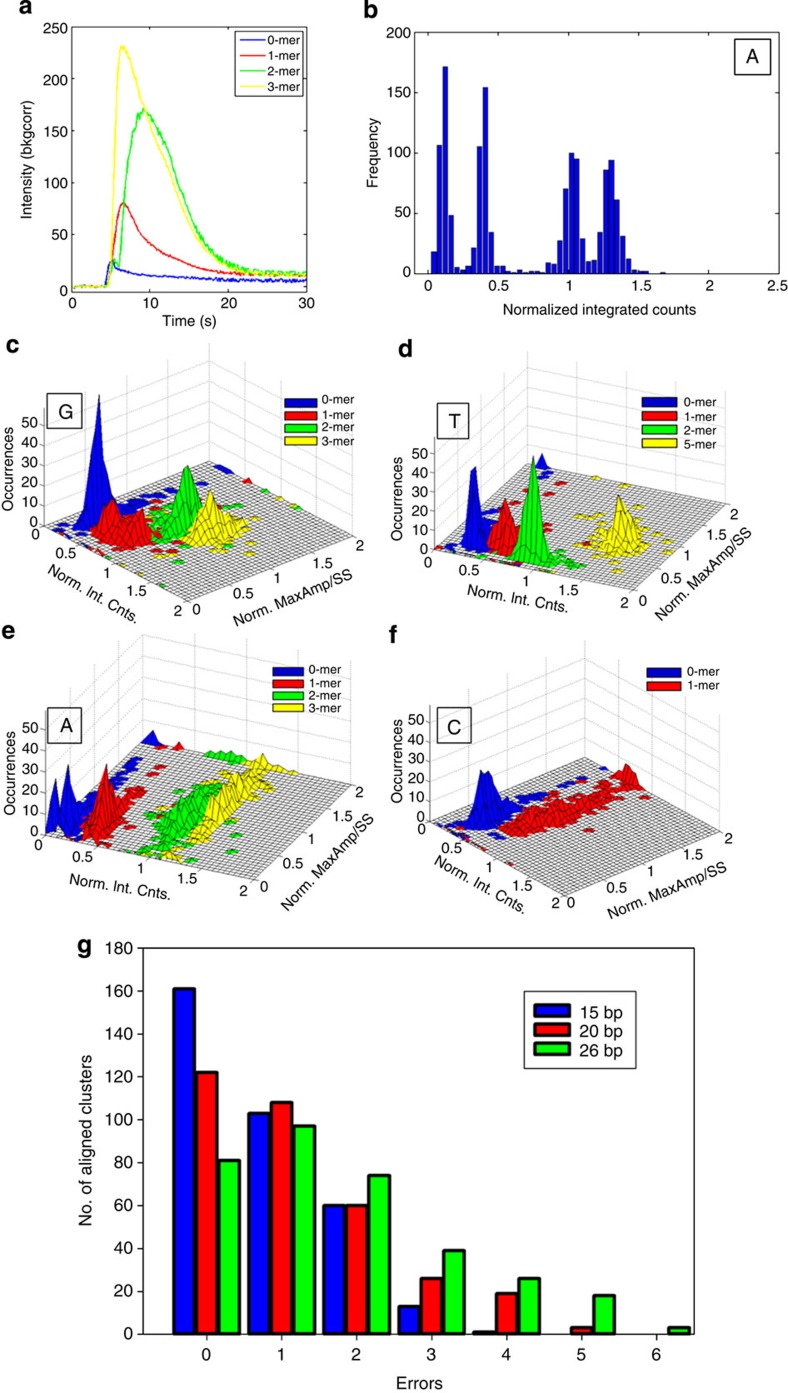
Homopolymer and base calling in SPDBK. (**a**) Averaged time traces for flows 3, 7, 15, 23 that correspond to 0, 1, 2 and 3-mer ‘A’ flows. (**b**) Histograms of all 11 ‘A’ flows from a 44 flow sequence. Histograms are representative of homopolymer repeats. K-means clustering method was used to discriminate clusters and perform base calling on homopolymer repeats. When MaxAmp/SSAmp and integrated counts are binned, three-dimensional histograms for the two axes are used to demonstrate the additional discrimination power for homopolymer discrimination of G, A, C and T flows. (**c**) Three-dimensional (3D) histograms of G homopolymer repeats from flows 5, 25, 29 and 37, which correspond to homopolymer repeats of 0, 1, 2 and 3-mers, respectively. (**d**) 3D histograms of T homopolymer repeats from flows 4, 8, 12 and 20, which correspond to homopolymer repeats of 1, 0, 5 and 2-mers, respectively. (**e**) 3D histograms of A homopolymer repeats from flows 3, 7, 15 and 23, which correspond to homopolymer repeats of 0, 1, 2 and 3-mers, respectively. (**f**) 3D histograms of C homopolymer repeats from flows 6 and 14, which correspond to homopolymer repeats of 0 and 1-mers, respectively. (**g**) After 44 flows, a random ROI with 357 clusters was chosen and base calling was performed as described in the text. Of the 357 clusters, 336 clusters were successfully aligned, which accounts for 91% of the clusters. Of these 91% aligned reads, the error distributions are shown for the following minimum overlap lengths: 15 bp (blue bars), 20 bp (red bars) and 28 bp (green bars).

From our previous theoretical kinetics (equations (4) and (5)), simulation (Fig. 2) and experimental studies (Figs 3 and 4), we determined that the number of photons emitted from a cluster is likely correlated to the stability of the signal complex at a certain flow. These results implied that the polymerase may have longer dwell times for homopolymer repeats, whereby more nucleotides are incorporated. The longer dwell time of the polymerase correlates with more photons observed at the cluster, which was clearly observed for the averaged time traces from representative 0−, 1−, 2− and 3−mer ‘A’ flows (Fig. 6a). Thus, we implemented integrated photon counts as a rudimentary base-calling feature and to discriminate homopolymer repeats.

Since the integrated photon counts from a single cluster also depends on the number of templates in that cluster, we normalized the cluster’s integrated counts by estimating the cluster template numbers (see Methods for further explanation). For future sequencing efforts, we anticipate that this step can be achieved in heterogeneous template sequencing by including a ‘calibration’ sequence at the beginning of each template. To demonstrate the efficacy of homopolymer calling, we normalized integrated counts at each cluster for all ‘A’ flows. We binned the integrated counts and clearly resolved four distinct peaks (Fig. 6b).

Previously, we showed that the variable MaxAmp/SSAmp can also be implemented as a potential base discrimination axis for improved base calling (Fig. 4b,d). We used this second discrimination axis and show the bivariate sequencing data (Max Amp/SS Amp and integrated counts) to demonstrate the additional discrimination power for homopolymer discrimination of G, A, C and T flows (Fig. 6c–f)). On the basis of the bivariate distributions for all of the flows (Supplementary Fig. 6), we chose appropriate thresholds and implemented a two-dimensional K-means clustering method for the bivariate sequencing data to perform base calling at each cluster independently on the randomly chosen population of 357 clusters.

Since we are using a cyclic sequencing approach, we predicted the average read length for 44 flows of cyclic sequencing is about 28 bp (assuming 0.63 bp per flow, see Supplementary Information, Estimating Speed of Cyclic Sequencing for detailed description). Using the known sequence as a reference, we applied a general alignment algorithm to determine the efficacy of our base-calling methodology for our sequencing method. Of the total 357 reads, we determined 336 clusters were successfully aligned, which accounts for 91% of the clusters. Of these 91% aligned reads, we determined the error distributions when we set the minimum overlap length to 15, 20 and 28 bp (Fig. 6g). As the read length increases, more errors accumulate, due to the deterioration of the signal at later flows. When considering the 44 flow experiment and the full-length alignment, a detailed breakdown of the specific errors provides a clearer picture of the source of the majority of the errors, which is further discussed below (Supplementary Fig. 6). We have achieved reliable DNA sequencing results by using polymerase/DNA-binding kinetics on DNA clusters.

### ΦX174 genome sequencing using SPDBK

Preliminary sequencing data is demonstrated using a ΦX174 sample library, where each cluster is generated from a different strand. In total, 64 flows are sequentially introduced into the flow cell. With each flow, a portion of the cluster intensity signals are integrated for base calling. Integrated intensities for individual clusters were classified using a Gaussian Mixture Expectation-Maximization method. The integrated cluster intensities are classified into multiple classes, which represent zero-base-incorporated, 1-base-incorporated, 2-base incorporated and so on.

The generated reads are clipped at 20 bp. Approximately 5,000 reads in a single FOV (Supplementary Data 1) are aligned to the ΦX174 reference genome using Bowtie sequence aligner (Table 1, Supplementary Data 2). To demonstrate the accuracy of match versus mismatch discrimination, 0 versus 1-mer base calling was performed and the results (Supplementary Data 3) were aligned to a synthetic homopolymer-free ΦX174 reference genome (Supplementary Data 4). For comparison, an equivalent number of random reads are generated and aligned to the ΦX174 reference genome using Bowtie sequence aligner (Table 1).

The rudimentary base-calling method (integrated counts), achieved reasonable raw accuracy of 95% for non-homopolymer regions and 90% with homopolymer regions at 20 bp read lengths. We expect that further improvement of the sequencing quality will be achieved through improvements in the base-calling algorithm, enzyme engineering and fluidic delivery as well as surface and DNA secondary structure studies.

## Discussion

By monitoring polymerase/DNA-binding kinetics, we have shown that the incorporation of correct nucleotides by the polymerase can be differentiated from the mismatch nucleotides in bulk solution as well as on a sequencing flow cell. We further demonstrated DNA sequencing using this concept with one single template and a ΦX174 genome. While this study was carried out using an unengineered polymerase, minimal formulation optimization, syringe-pump-based fluidics system and a rudimentary base-calling algorithm, we clearly proved that this chemistry has distinctive advantages. Labelling the polymerase instead of the nucleotides drastically simplifies signal processing, detection and labelling complexity by reducing the reporter signal from four signals to a single signal. In this study, fluorophore was used to label the polymerase and hence an optical system was employed for detection. It is conceivable to label the polymerase with a variety of nanoparticle probes to allow alternative detection schemes on a sensor chip^22^,^23^. It is also possible to use the intrinsic properties of the polymerase, such as charge and size, as the readout for label-free detection on a semiconductor sensor chip^24^,^25^,^26^. The other unique advantage of this chemistry is that it combines the advantages of Illumina’s integrated workflow with Ion Torrent’s fast turnaround sequencing.

Most technologies currently in the market have been refined for over the past 10 years. This study is the initial proof-of-concept of a new method and in the following sections we discuss how the technical limitations of speed, accuracy, and cost can be overcome.

Accuracy is one of the most important sequencing metrics especially for clinical sequencing. We implemented a very rudimentary sequence alignment to provide a detailed breakdown of our data and the error sources on a cluster by cluster basis (Supplementary Fig. 6), in addition to comparative alignment statistics on a library of ΦX174 reads with and without homopolymer calling. Our basic alignment data reveal the subtle deficiencies of our method, that is, homopolymer-calling deficiencies and sequence context errors (Supplementary Fig. 6). To improve accuracy, we propose to implement existing algorithms to correct for phasing that is known to cause errors in synchronized sequencing approaches^27^. Additional base-calling improvements, such as Bayesian learning algorithms^28^, can be implemented to capture kinetic patterns that may exist as a result of sequence context.

As was suggested earlier in our rudimentary base-calling algorithm, different template density in each cluster resulted in amplitude and duration variations. We can correct for some of this variability by including calibration sequences as part of the adapter sequences to estimate actual template density. Base-calling errors can be introduced by variability in polymerase/DNA-binding kinetics, which is influenced by factors such as DNA secondary structure^29^,^30^,^31^ and local cluster environments. Dimethylsulphoxide, Betaine and elevated temperature can help minimize DNA secondary structure^29^,^30^,^31^, which can relieve the impact secondary structure may have on the reproducibility of polymerase/DNA-binding kinetics^32^. Since three-dimensional local cluster environments on existing substrates can confound the polymerase/DNA-binding kinetics, we can implement formulation and surface feature modifications to minimize the variability. While these factors are paramount for improved performance of this sequencing chemistry, we believe that we can also rapidly evolve this sequencing chemistry using well-established polymerase engineering and fluidics solutions.

Polymerases have been successfully engineered on multiple occasions to improve their performance over their native counterparts^33^,^34^,^35^. We achieved proof-of-concept for this sequencing chemistry with wild-type mesophilic Bsu polymerase and minimal tuning of formulation conditions. Moving forward, polymerase engineering combined with formulation optimization could address many of the sequence viability questions. For example, it may be possible to engineer a mutant polymerase to have high fidelity, relatively long incorporation time (*k*_cat_) for correct nucleotides and fast DNA off-rate (*k*_−1_) once mismatch nucleotides are encountered. This would increase base discrimination and improve homopolymer accuracy significantly. By using a thermostable polymerase and sequencing at elevated temperatures (current Illumina protocol) the impact of DNA secondary structure and sequencing context will be alleviated. One other approach to improve the accuracy is to use labelled nucleotides and an unlabelled processive polymerase^36^,^37^. In this case, the variability could be reduced as the signal complex is the ternary complex alone, removing the binary complexes from equations (4) and (5).

The use of natural nucleotides allows very fast sequencing chemistry since polymerases incorporate natural nucleotides with a speed of ten to hundreds of bases per second^16^. The speed of our sequencing chemistry is simply limited only by the fluidics. Due to the syringe pump and flow cell design, the flow profiles for each individual cycle not only vary from flow to flow, but also across the FOV. These variations introduce asynchronization of flow and mixing across clusters and cycles that eventually flattens the signal, and in extreme instances, leads to false negatives. Therefore, a pressurized system and valving similar to those used in pyrosequencing methods would likely improve the accuracy through improved uniformity of the flow and mixing^7^. More recently, pyrosequencing was performed using digital microfluidics^38^, which is an alternative method to insure fast mixing, uniformity of the flow and synchronization.

Our current cycle time for this sequencing chemistry includes 20 s chemistry time, 5 s wash time and 20 s delay for pumping time. By implementing pressurized fluidics, pump times become limited by flow rate, which can be estimated from the pressure of the system, geometry of flow cell and tubing and the valve response times. Using solenoid valves with response times around 30 ms (The Lee Company), tubing diameter of ~400 mm, channel length=60 mm, pressure held at 20 p.s.i. and aqueous based solutions, pump times of <0.5 s can be achieved. With improved/engineered polymerase and formulation optimization, it is conceivable that we can reduce chemistry times to <2 s for a small ensemble of 1,000–2,000 template molecules^16^. Combining wash steps with faster fluidic exchange time (<2.5 s) and assuming 0.63 bp per cycle (see Methods), we believe the cycle times for this chemistry could readily approach 7–15 s.

We have also demonstrated excellent base discrimination on low-cost optical instrumentation (Supplementary Fig. 7), which makes this platform highly scalable^39^,^40^. By combining the ease and speed of Illumina’s clustering chemistry (5.0 × 10^5^ clusters m^−2^ in <1 h), cycle times of 45 s, the scalability associated with large FOV (>9 mm^2^) low-cost fluorescence imaging instruments^39^,^40^ and attainable read lengths of 100 bp, the throughput of this instrument would be around 5 Gbp with a TAT, which includes cluster amplification of 1.5 h. While we recognize the impact of lower cost optics (lower NA), we can compensate for subsequent diminished signal to noise with three separate approaches. We can increase the number of templates/cluster, increasing signal/cluster. As demonstrated in the work, the basic base-calling method relies only on the ‘integrated photon counts’ as the main discrimination signal. This is possible because the much lower signal amplitude when mismatch nucleotides are present. It is possible, then, to reduce the data collection rate as fast frame rate real-time monitoring is not required. Without the requirement for real-time data collection, we envision the possibility to further increase the throughput with fast fluidic coupling, lower signal-to-noise ratio and larger FOV. Finally, improving the fluidics to increase synchronization of enzyme delivery to templates will also improve our SNR.

While the sequencing results suggest homopolymer discrimination is challenging, TBRS may not require high hompolymer accuracy as many gene panels do not have long homopolymers (Supplementary Table 1). Taken together, this new sequencing chemistry has the potential to deliver the accuracy of ensemble approaches^1^,^7^,^41^, the read lengths of natural biochemistry sequencing technologies^27^,^41^, fast TAT that fits clinical apps^42^ and ease of sample preparation using Illumina’s bridge amplification^1^, which positions as a competing platform for TGRS applications.

## Methods

### Instrumentation

Data were collected on a modified Genome Analyzer I from Illumina Inc. (San Diego, CA). This instrument has been fully described in detail elsewhere^1^. Slight modifications were made to accommodate the synchronized sequencing scheme. In addition, optical components were modified to improve the collection efficiency for Cy3 dye that was chosen for these experiments. The filter turret was modified to house a single 540 LP filter. The imaging set-up consisted of a modified GA instrument (Supplementary Fig. 4) whereby the excitation sources was 532 nm, the flow cell was total internal reflection fluorescence illuminated, the power density was about 1/10 of the standard GA imaging power density, the emitted light from the DNA clusters was collected through a × 20 Nikon Objective (NA=0.75) and imaged onto a Hamamatsu ORCA-Flash 2.8 CMOS through a 532 LP filter. The incident power density was approximated to be 0.1 W cm^−2^. Data were typically collected at 10 frames s^−1^ for 40 s, unless otherwise noted. Samples were introduced with a flow rate of ~4 ml min^−1^ using the GA syringe pump configuration.

### Flow cell preparation

Cluster amplification was performed according to the manufacturer’s protocol using paired end cluster chemistry and paired end flow cells V4. Template concentrations were determined to achieve a cluster density of ~20,000 clusters mm^−2^. All calibration experiments to optimize sequencing conditions (NaCl and dNTP titration experiments) were performed using a template with the following insertion sequence: 5′- CTAAGTTTTTCACTTAAAGAGGCTTAGGGAAAGTGATTTTTAAAGAGTCACTGTTACATGGTAATATGCCGTTCA -3′. This template was bridge amplified to create a monotemplate flow cell.

### Expression and purification of BSU Pol I

The Pol I gene from *Bacillus subtilis* was codon optimized and purchased from DNA2.0. The gene was then PCR amplified and subcloned into a pET15b vector containing an amino-terminal (N-terminal) 6xHIS tag followed by a thrombin cleavage site (MGSSHHHHHHSSGLVPRGSH). Site-directed quikchange mutagenesis was performed to replace the second serine following the 6xHIS tag with a cysteine (MGSSHHHHHHSCGLVPRGSH). This construct was used to express BSU polymerase with an N-terminal 6xHIS tag and unique exposed cysteine residue for maleimide chemistry labelling. Purified protein was then concentrated to 100 μM and conjugated to Cy3-Maleimide reactive dye (GE) using the manufacturers protocol. Detailed protein purification and labelling protocols are described below.

### Expression and purification of BSU Pol I

The Pol I gene from *Bacillus subtilis* was codon optimized and purchased from DNA2.0. The gene was then PCR amplified and subcloned into a pET15b vector containing an N-terminal 6xHIS tag followed by a thrombin cleavage site (MGSSHHHHHHSSGLVPRGSH). Site-directed quikchange mutagenesis was performed to replace the second serine following the 6xHIS tag with a cysteine (MGSSHHHHHHSCGLVPRGSH). This construct was used to express BSU polymerase with an N-terminal 6xHIS tag and unique exposed cysteine residue for maleimide chemistry labelling. The pET15b-BSU was confirmed by sequencing and transformed into BL21 Star (DE3) expression cells from Invitrogen. The transformed cells were cultured at 37 °C in 2.8 l Fernbock flasks until an OD600 of 0.8 was reached. Protein expression was then induced by addition of 1 mM isopropyl-β-D-thiogalactoside, followed by 3 h of additional growth. The cultures were then centrifuged at 7,000 r.p.m. for 20 min. Cultures of 4 l typically yielded 25 g of wet cell pellet. Cell pellets were stored at −80 °C until purification.

Bacterial cell lysis was performed by resuspending the frozen cultures in 10 × w/v lysis buffer (Tris pH 8.0, 500 mM NaCl, 1 mM EDTA, 1 mM DTT). EDTA-free protease inhibitor (Roche) was added to the resuspended cell pellet. All lysis and purification steps were performed at 4 °C. The resuspended culture was passed through a microfluidizer four times to complete cell lysis. The lysate was then centrifuged at 20,000 r.p.m. for 20 min to remove cell debris. Polyethylenimine (final concentration 0.5%) was added to the supernatant slowly with stirring for 45 min to precipitate bacterial nucleic acid. The lysate was centrifuged at 20,000 r.p.m. for 20 min; the pellet was discarded. The lysate was then ammonium sulfate precipitated using two volumes of cold saturated (NH4)_2_SO4 in sterile dH_2_O. The precipitated protein was centrifuged at 20,000 r.p.m. for 20 min. The protein pellets were resuspended in 250 ml of Buffer A (50 mM Tris pH 8.0, 300 mM NaCl, 20 mM imidazole, 1 mM EDTA, 1 mM DTT). The resuspended lysate was then purified using a 5 ml HisTrap FastFlow column (GE) pre-equilibrated in buffer A. The column was eluted using a 100 ml gradient from 20 mM to 1 M imizadole. Peak fractions were pooled and diluted with buffer C (Tris pH 7.5, 1 mM EDTA, 1 mM DTT) until the conductivity was equal to buffer D (Tris pH 7.5, 50 mM NaCl, 1mM EDTA, 1 mM DTT). The pooled fractions were then loaded onto a 5-ml HiTrap Heparin Fastflow column. The polymerase was then eluted using a 100 ml gradient from 50 mM to 1 M NaCl. Peak fractions were pooled and concentrated before fluorescent labelling.

### Labelling BSU Pol I with Cy3-Maleimide

Purified BSU Pol I was buffer exchanged into conjugation buffer (50 mM ACES pH 7.4, 20 mM NaCl, 0.2% Tween-20) using illustra NAP G-25 columns (GE). The protein was then concentrated to 100 μM and conjugated to Cy3-Maleimide reactive dye (GE) using the manufacturer’s protocol. The labelling reaction was incubated at 4 °C for 16 h, followed by diafiltration and concentration using vivaspin 6 (30 kDa MWCO) concentrators (GE). Final buffer exchange and excess Cy3-Maleimide removal was performed using illustra NAP G-25 columns pre-equilibrated in storage buffer (50 mM ACES pH 7.4, 20 mM NaCl, 0.2% Tween-20, 1 mM DTT). Molar labelling efficiency was calculated spectrophotometrically using extinction coefficients of 150,000 M^−1^ cm^−1^ and 55,810 M^−1^ cm^−1^ for Cy3 and BSU Pol I, respectively. Protein lots with labelling efficiencies of ≥95% were aliquoted and flash frozen in liquid N_2_ and stored at −80 °C until use.

### Enzyme validation

Enzyme activity was determined by burst assay^43^. In brief, 200 nM of enzyme (by Bradford assay) was preincubated with 1,000 nM duplex DNA in reaction buffer (10 mM Tris pH 8.0, 50 mM NaCl, 10 mM MgCl2, 1 mM DTT). Duplex DNA was constructed by annealing 1:1.1 molar ratios of primer (5′-Cy5- GCTTGCACAGGGCCTCGAC -3′) and template.

(5′- CGTTAGTAAGGTCGAGGCCCTGTGCAAGC -3′) oligonucleotides (IDT). The enzyme–DNA complex was then rapidly mixed with 100 μM dCTP for various times from 0 to 2 s at 37 °C using a RQF-4 Rapid Quench Flow (KinTek Corp.) Reactions were quenched by addition of 500 mM EDTA. Product formation (*n*+1) was separated from substrate (*n*) by 15% denaturing polyacryalamide gel electrophoresis. Products were visualized using a Typhoon imager and quantified using ImageQuant TL (GE) and Grafit 7.0 (Erithacus). Only enzyme lots possessing ≥90% activity by burst assay were used in subsequent pre-steady-state and GA analysis.

### Pre-steady-state analysis

The nucleotide concentration dependence of product formation was determined by rapid-quench analysis using a RQF-4 Rapid Quench Flow (KinTek Corp.). A preincubated complex of 1000, nM enzyme and 200 nM duplex DNA was rapidly mixed at 45 °C with various concentrations of dCTP in high salt reaction buffer (20 mM ACES pH 7.4, 300 mM NaCl, 1 μM acetylated bovine serum albumin (BSA; Ambion), 10 mM MgSO_4_, 1 mM TCEP). Reactions were quenched by addition of 500 mM EDTA and quantified as previously described. The assay was repeated using a mismatched dATP:dG for extension. Mismatched product formation required nucleotide concentrations from 100 to 3,000 μM and longer reaction incubation times up to 60 min to observe product formation. The product formation at each concentration nucleotide concentration was fit by non-linear regression to a single-exponential equation (product=*Ae*^*kt*^+*C*). The nucleotide concentration dependence of the resulting rates (*k*) were then fit to a hyperbolic function (*k*_observed_=*k*_max_X[S]/*K*_d,app_+[S]).

The effects of NaCl and nucleotide concentration on transient enzyme–DNA binding were observed using fluorescence stopped flow techniques. A preincubated complex of 600 nM fluorescently labelled enzyme and 100 μM dCTP was rapidly mixed with duplexed DNA in high salt reaction buffer (20 mM ACES pH 7.4, 1 μM acetylated BSA, 0.02% Tween-20, 10 mM MgSO_4_, 1 mM TCEP). The final NaCl concentration of the buffer was varied from 62.5 to 325 mM. The duplexed DNA was assembled by annealing the previously described template oligo with a primer containing a fluorescein modified thymidine (shown underlined) five bases from the incorporation site (5′- GCTTGCACAGGGCCTCGAC -3′). Fluorescein is an environmentally sensitive dye that is quenched on protein interaction^44^.

The transient enzyme–DNA association was monitored by excitation of fluorescein at 495 nm and fluorescent emission using a 520 nm high-pass filter. The nucleotide concentration dependence on transient enzyme–DNA binding was observed by preincubating 600 nM of fluorescently labelled enzyme with various concentration of dCTP from 0 to 80 μM in high salt reaction buffer containing 300 mM NaCl. The reaction was started by rapid mixing with 400 nM fluorescein labelled DNA. All concentrations were final after mixing.

### Base discrimination optimization on GA

On the basis of the pre-steady-state analysis, the nucleotide concentration dependence of product formation was determined by titrating nucleotide concentration under a high salt condition. The effects of NaCl and dNTP on base (correct versus mismatch) and homopolymer discrimination (_max_ amplitude/steady-state amplitude) were determined by performing titrations of [dNTP] or [NaCl] while holding the other constant. From pre-steady-state analysis, the [NaCl] titration was performed by holding the [dNTP] fixed at 100 μM, which was determined to be the peak concentration before misincorporation becomes problematic for dCTP. To demonstrate a qualitative correlation between stopped flow results, the [NaCl] titrations were performed over the range of 62–375 mM using [dCTP] as the correct nucleotide and [dGTP] as the mismatch nucleotide. To optimize the reaction conditions for nucleotide concentration, the reactions were performed at 300 mM NaCl and the correct and mismatch nucleotide concentrations were varied from 5 to 500 μM.

Reactions were performed by pumping 250 μl of reaction buffer with the correct (dCTP) or mismatch nucleotide (dGTP) at 300 μM through the flow cell. In addition to the respective nucleotides, the reaction buffer components also included the following: 50 mM ACES pH 7.4, 1 μM acetylated BSA, 0.02% Tween-20, 10 mM MgSO_4_, 1mM TCEP, 125 nM SSB (Epicentre), 2 mM CaCl_2_, 100 nM glucose oxidase, 1.5 μM catalase, 56 mM glucose. Two 250-μl wash cycles were introduced into the flow cell after the reaction mix. The wash 1 buffer components included the following: 50 mM ACES pH 7.4, 1 μM acetylated BSA, 0.02% Tween-20, 2.5 mM EDTA, 300 mM NaCl. Wash 2 buffer components included the following: 250 mM ACES pH 7.4, 1 μM acetylated BSA, 0.02% Tween-20, 1 mM TCEP, 125 nM SSB (Epicentre), 100 mM NaCl.

### Sequencing using polymerase DNA-binding kinetics

Sequencing reactions were run using a mixture of synthetic templates with the following insertion sequences: templates were mixed to insure approximately equal cluster numbers for each respective template. Sequencing reactions were run on the previously described modified GA instrumentation and analysis was performed as described below. Since we are using a synchronized sequencing scheme, the nucleotides were introduced into the flow cell using the following sequencing ‘G’, ‘T’, ‘A’, ‘C’ with the following concentrations: 100, 300, 100 and 100 μM, respectively. In addition to the respective nucleotides concentrations and 350 mM NaCl in the reaction buffer, the final sequencing reaction buffer formulations (reaction buffer, wash 1 and wash 2) were identical to those described in the base discrimination assays above.

### ΦX174 sequencing

ΦX174 Control v3 (FC-110-3001) was purchased from Ilumina. The Φx library was prepared according the to the manufacturer’s instructions. A ΦX174 library was loaded onto the flow cell at a concentration of 0.8 pM and clustered using a cBot according the manufacturer’s instructions with the following modification: the sequencing primer solutions were swapped a solution that was composed of an oligo (5′- CGGCGACCACCGAGATCTACACTCTTTCCCTACACGACGCTCTTCCGA -3′) dissolved in HT-1 buffer at a final concentration of 500 pM. The flow cell was mounted on the GA as previously described and 200 μl solution composed of 50 mM ACES pH 7.4, 100 mM NaCl, 0.02% Tween-20, 1 mM TCEP, 1 μM acetylated BSA, 4 ng μl^−1^ SSB, 2 mM MgCl_2_, 50 nM Cy3-Klentaq, 25 μM dATP, 25 μM dGTP and 25 μM dTTP was flowed into the lane and incubated at 47 °C for 10 min. The presequencing run mix was washed with a high salt wash buffer (Buffer 1) composed of 50 mM Tris pH 7.5, 1 M NaCl, 0.02% Tween-20 and 0.1 mM EDTA. The sequencing run consisted of cycling through following reagents: (1) Buffer 1, (2) Buffer 2, (3) Buffer 3, (4) Buffer 4 (5) Buffer 4+Enzyme Buffer 2 was composed of 50 mM ACES pH 7.4, 300 mM NaCl, 0.02% Tween-20, 1 mM TCEP, and 1 μM acetylated BSA. Buffer 3 was composed of 50 mM TrisHCl pH 7.4, 40 mM NaCl and 4 mM CaCl_2_ and 750 mUnits apyrase (NEB, M0393L). Buffer 4 was dNTP-specific. Buffer 4 for either 1 mM dATP or 1 mM dTTP was composed of 50 mM ACES pH 7.4, 300 mM NaCl, 0.02% Tween-20, 1 mM TCEP, 0.1 × acetylated BSA, 2 mM MgCl_2_ and 4 ng μl^−1^ SSB. Incorporation buffer was composed of Buffer 4 with the respective dNTPs and the inclusion of 75 nM Cy3-BSU.

The corresponding volumes (μl) and flow rates (ml min^−1^) for the aspiration of each solution (fluidic scheme was in a pull configuration) were the following (1) 240 and 3.2, (2) 150 and 3.2, (3) 240 and 3.2, (4) 200 and 3.2 and (5) 240 and 6.4. The dispense rate of the syringe pump was 7 ml min^−1^. The total fluidic exchange times are equivalent to 27 s. The incident power density was approximated to be 0.1 W cm^−2^. Data were typically collected at 10 frames s^−1^ for 40 s, unless otherwise noted.

### Software and computation

Data processing pipeline consists of three steps: spot detection, time series extraction and base calling. In spot detection, the locations of the clusters were determined on an ‘average image’, which were computed by averaging the first 100 frames of the TIFF movie collected. The averaged images were filtered with a Difference of Gaussian filter, with a centre s.d. of 0.7 pixels and a surround s.d. of 2 pixels. Centre pixel locations of clusters were defined as the regional maximum on the filtered image on a four-connected neighbourhood. Within a five-by-five pixel neighbourhood around the centre pixel, background pixels were chosen, which are defined as pixels whose Difference of Gaussian response is less than the Otsu’s threshold and more than two pixels away from the centre pixel. For each cluster, a variable number of background pixels were chosen.

When extracting time series from each cluster, each frame of the movie was filtered with a Guassian filter with a s.d. of 1 pixel. The intensity value at each centre pixel is extracted as the foreground intensity. The background intensity of a cluster is extracted as the mean of background pixels’ intensities (Supplementary Fig. 2).

For each flow, each cluster is basecalled using the background-subtracted signal. For base calling, the DC biases of the individual time traces are removed by subtracting off the average intensities before each flow, which is computed by averaging intensities at the first 40 frames. The cluster intensities are integrated between a time window that is related to the incorporation speed between bases, that is, the first 150 frames for G and A flows, the first 200 frames for C and the first 350 frames for T. Since the integrated photon counts from a single cluster also depends on the number of templates in that cluster, the cluster integrated counts are normalized by the sum integrated counts of certain flows. In this example, flow 7, 12, 20, 23 and 32 are chosen, because these flows include significant number of homopolymers, and thus the sum of all these flows is a more robust estimation of the cluster template numbers. We envision that this step can be achieved in inhomogeneous template sequencing by including a ‘calibration’ sequence at the beginning of each template.

### Estimating speed of cyclic sequencing

Without losing generality, we assume the following flow order GTAC and that all bases are equally distributed along a random template. We also assume the following percentages for n-mers in a bacterial genome, 75% monomer, 24.5% 2-mer and 0.5% 3-mer and longer. Starting from a successful flow of G incorporation (match), we deduce that the probabilities of the next matching bases are the following:


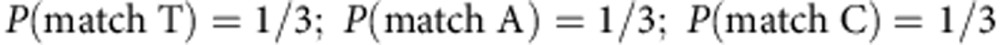


The total number of flows that are required to reach the next matching base can be calculated as follows:


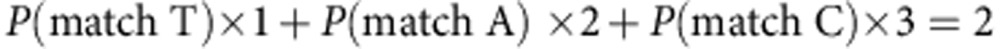


Taking into account the aforementioned estimated n-mer percentages for a genome, the estimated total number of bases incorporated in the next matching flow is expressed as follows:


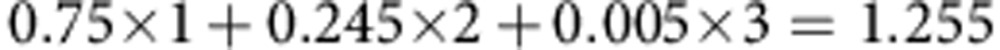


On average, the speed of cyclic sequencing can be estimated as 1.255 bp per 2flow≈0.63 bp per flow.

## Author contributions

M.J.R.P., C.Z. and M.M.H. conceived the study. M.J.R.P., M.K. and C.Z. performed the data analysis. C.Z., A.K. and J.V. performed signal and image processing. M.J.R.P., C.Z. and S.E. built and designed the instrumentation. M.J.R.P., M.K., R.P., C.-Y.C., J.S., B.W. and A.N. performed the kinetics and sequencing experiments. A.N., E.B. and C.G. performed protein purifications and conjugations. M.M.H., M.J.R.P., C.Z and M.K. wrote the paper. All authors contributed to meaningful discussions for this research.

## Additional information

**How to cite this article**: Previte, M. J. R. *et al*. DNA sequencing using polymerase substrate-binding kinetics. *Nat. Commun.* 6:5936 doi: 10.1038/ncomms6936 (2015).

## Supplementary Material

Supplementary Information Supplementary Figures 1-7 and Supplementary Table 1

Supplementary Data 1 The fastq file for the PhiX sequencing results

Supplementary Data 2 The *.sam file for the PhiX sequencing results

Supplementary Data 3 Contains the fastq file for the synthetic monomer PhiX sequencing results.

Supplementary Data 4 The *.sam file for the synthetic monomer PhiX sequencing results.

## Figures and Tables

**Table 1:** ΦX174 sequencing results.

**Data**	**Total reads**	**bp**	**# Aligned (%)**	**Mismatch rate**
Homopolymer	5,140	20	397 (7.7)	9.96% (58 perfect reads)
Random	5,140	20	1 (0.02)	—
Non-homopolymer	3,127	20	828 (26.5)	4.9% (453 perfect reads)

All raw reads and alignment results can be found in the respective *.fastq and *.sam files that are provided in the Supplementary Information.
