# Supplementary material for: DNA sequencing using polymerase substrate-binding kinetics
Source: Nat Commun. 2015 Jan 23;6:5936. doi: 10.1038/ncomms6936 (PMC4354037; doi:10.1038/ncomms6936)
Supplement: Supplementary Information — Supplementary Figures 1-7 and Supplementary Table 1 [file ncomms6936-s1.pdf]

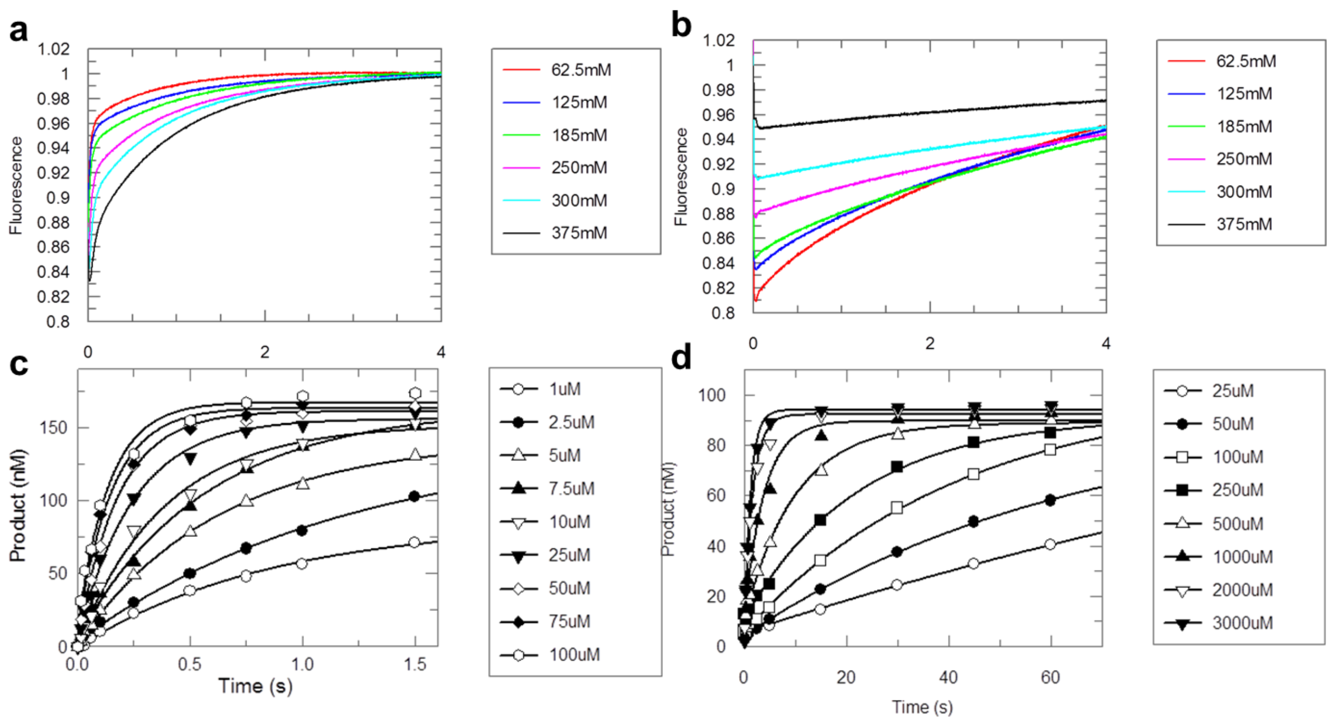

Supplementary Figure 1. Stopped flow nucleotide-induced fluorescence response shows NaCl concentration dependence for **(a)** correct (dCTP) and **(b)** mismatch (dATP) nucleotides. The nucleotides were rapidly mixed with BSU polymerase and FAM labeled 19/36mer in the presence of various NaCl concentrations. Quench flow nucleotide concentration dependence of product formation under high salt conditions are shown with increasing concentrations of **(c)** correct (dCTP) or **(d)** mismatch (dATP) nucleotides. The nucleotides were rapidly mixed with BSU polymerase and 19/36mer in 300mM NaCl buffer. The resulting time dependence of product formation for each nucleotide concentration was fit to a single exponential equation to obtain a rate.

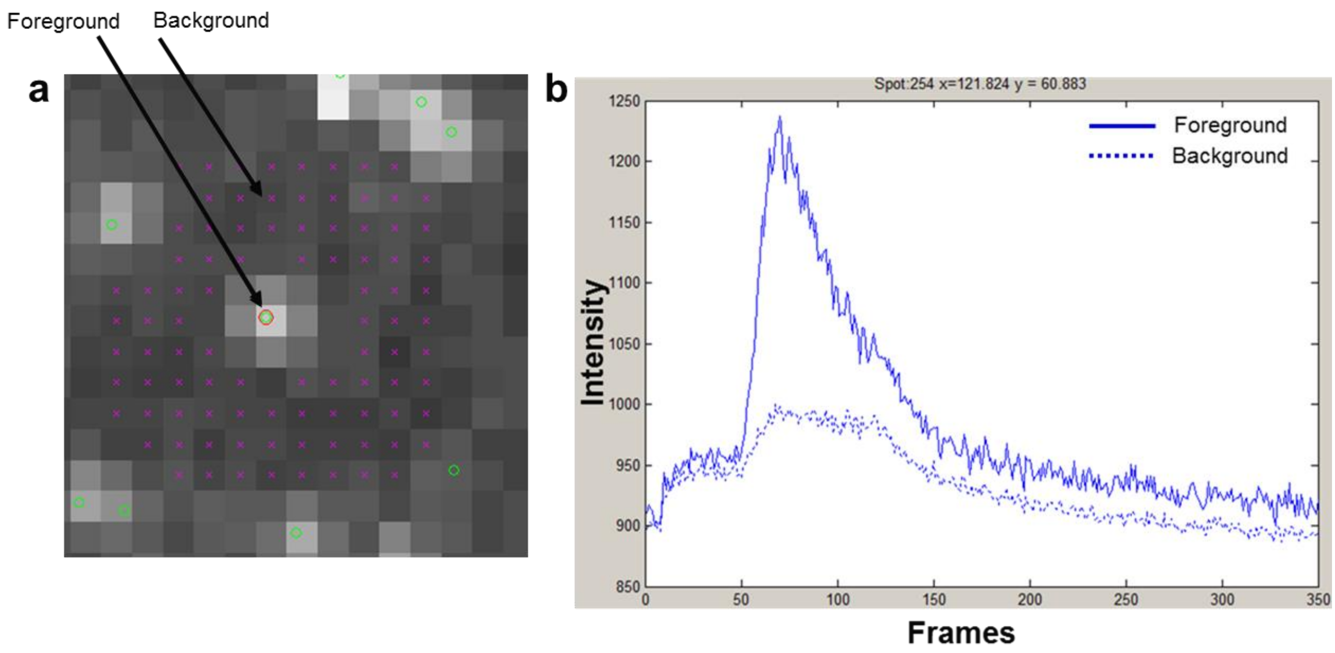

Supplementary Figure 2: (a) Average image from flow 12 with spot detection for both foreground and background. **(b)** Raw traces for foreground and background extracted from the image shown in (a).

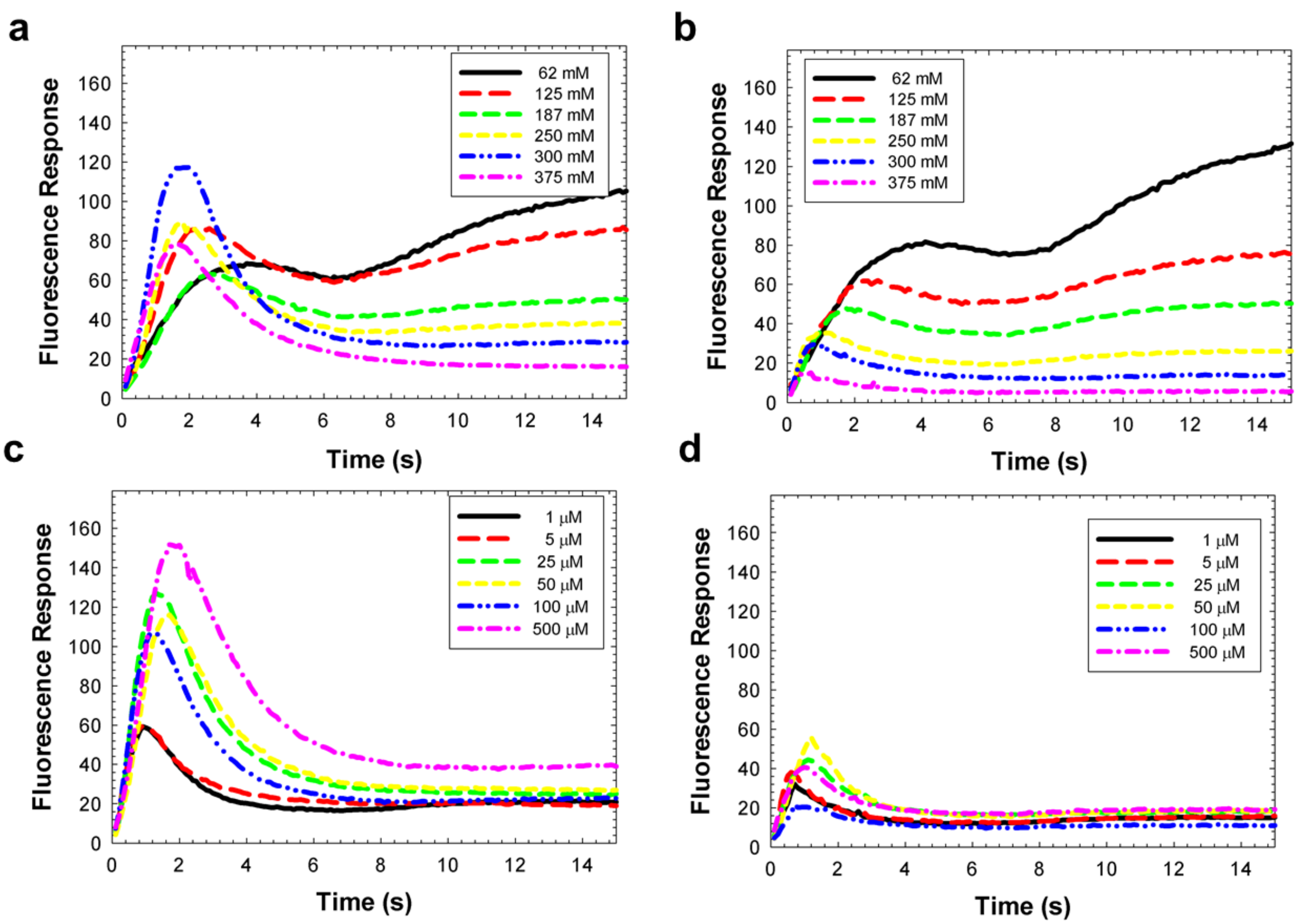

Supplementary Figure 3: In the presence of varying NaCl concentrations **(a-b)** or dNTP concentrations **(c-d)**. **(a, c)** Correct (dCTP) and **(b,d)** mismatch (dGTP) nucleotides and 200 nM BSU polymerase were introduced into a clustered flow cell that had underwent cluster amplification. Resultant time traces were background subtracted (**Supplementary Figure S2**).

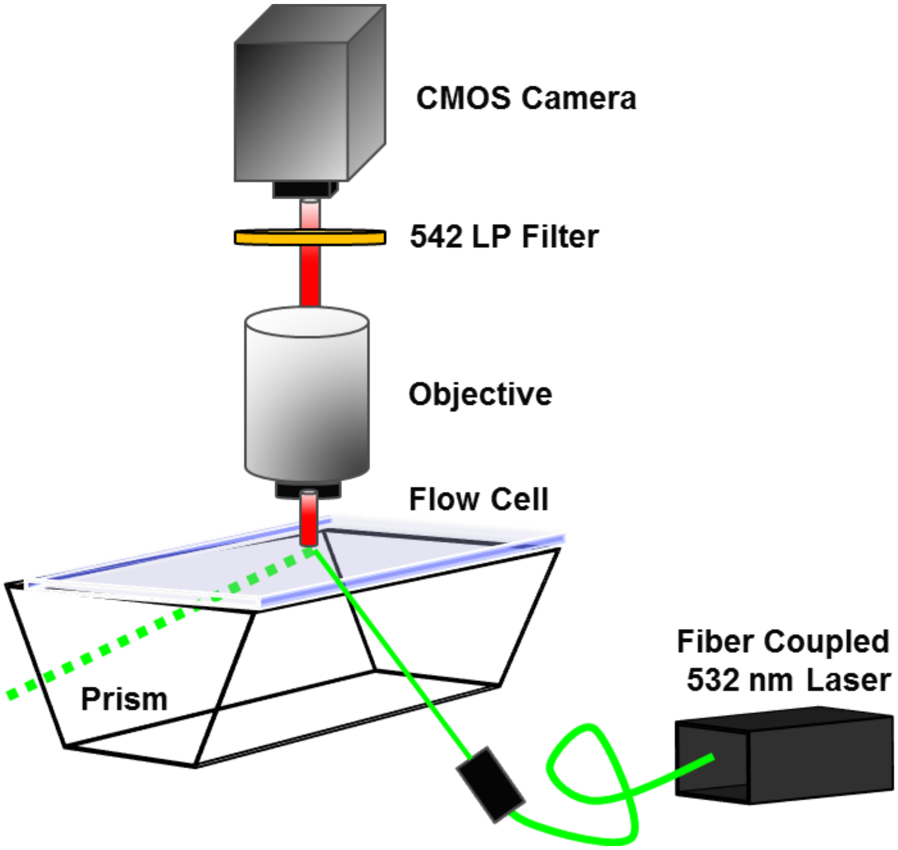

Supplementary Figure 4: Excitation light at 532 nm is fiber coupled. Fiber is positioned such that the light couple through the prism and sample to create a TIRF illumination field. Emitted light is collected through a 20 X Nikon Objective (0.75 NA) and a 542 LP filter. The sample is imaged onto a CMOS camera.

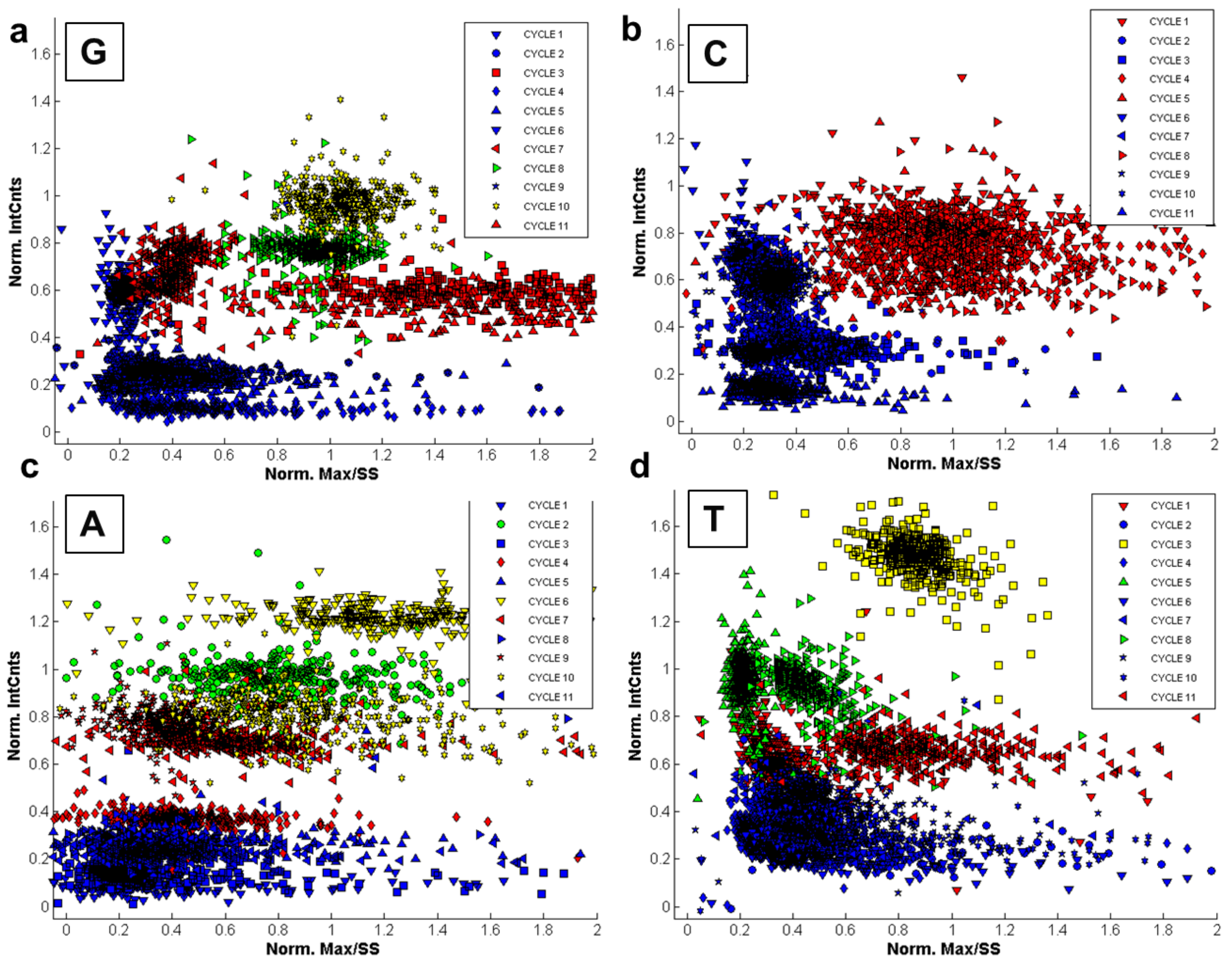

Supplementary Figure 5: Eleven individual 'G', 'A', 'T', and 'C' cycles were included in the 44 flow experiment. The integrated counts vs. Max Amp/SS Amp ratios for each of the 357 individual clusters chosen from a random ROI, are displayed in the 2D scatter plots. **(a)** 2D scatter plots for 'G' cycles correlate with flow #1, 5, 9, etc. of the 44 flow experiment. **(b)** 2D scatter plots for 'C' cycles correlate with flow #2, 6, 10, etc. of the 44 flow experiment. **(c)** 2D scatter plots for 'C' cycles correlate with flow #3, 7, 11, etc. of the 44 flow experiment. **(d)** 2D scatter plots for 'C' cycles correlate with flow #4, 8, 12, etc. of the 44 flow experiment. From these distributions, a K-clustering means method was implemented to set thresholds and perform base discrimination and homopolymer calling.

Position Consensus	1	2	3	4	5	6	7	8	9	10	11	12
Expected	C	T	-	A	A	-	G	-	-	T	T	T
# Correct	277	262	333	330	328	331	321	325	335	335	335	335
# Mismatch	1A/11T	-	1G	1T	-	4A/1T		11G	1C			
# Gaps (-)	47-	74-		5-	8-		15-			1-	1-	1-

Position Consensus	13	14	15	16	17	18	19	20	21	22	23	24
Expected	T	T	-	C	-	A	C	-	-	T	T	-
# Correct	335	335	332-	303	335	306	328	329	335	336	314	316
# Mismatch			4A		1C			7A	1A		2G	20G
# Gaps	1-	1-		33-		30-	8-				20-	

Position Consensus	25	26	27	28	29	30	31	32	33	34	35
Expected	A	A	A	G	A	-	G	G	-	C	-
# Correct	335	336	326	309	336	332	334	298	326	314	328
# Mismatch	1G			1T		3A/1T	2A		10G	1A/1G	8A
# Gaps			10-	26-				38-		20-	

Position Consensus	36	37	38	39	40	41	42	43
Expected	T	T	-	A	-	-	G	G
# Correct	335	277	334	336	277	333	336G	333G
# Mismatch		21A	1C/1G		37A/22T	3T		
# Gaps	1-	38-						3-

Supplementary Figure 6: Detailed breakdown of basecall error sources for 336 clusters aligned to the reference sequence for 28 bp.

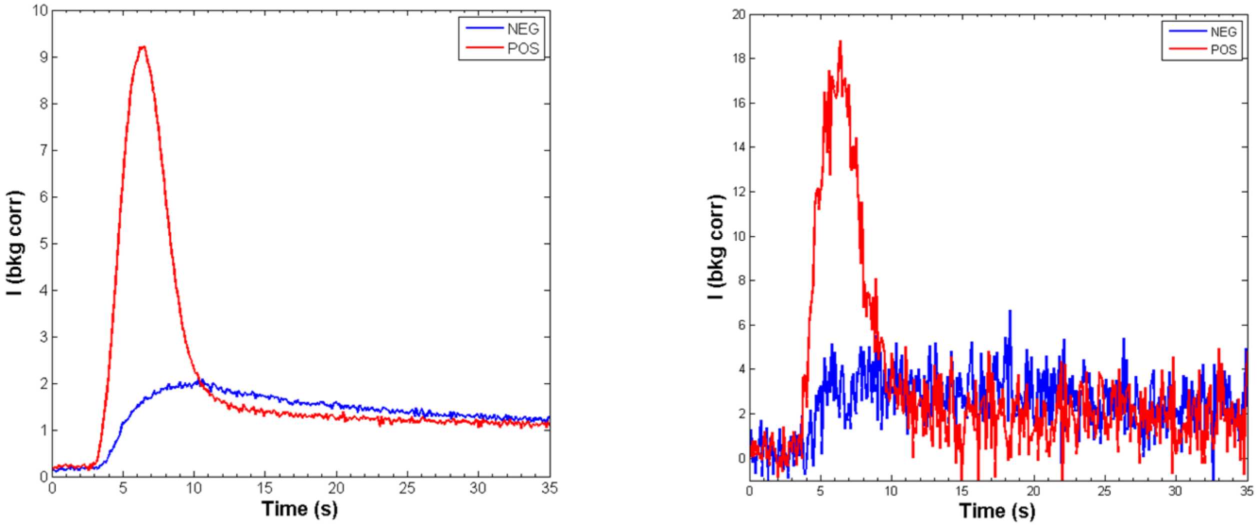

Supplementary Figure 7: Flow cell was illuminated with a low cost green LED (Luminex) that was collimated and passed through a 525/10 bandpass filter. The incident power at the sample plane was approximated as 0.1 W/cm^2. Emitted light is collected through a 10 X Olympus Objective (0.30 NA) and a 542 LP filter. The flow cell was imaged onto a low-cost Aptina CMOS 8 MP sensor. Correct, dCTP, and mismatch, dGTP, base discrimination and image processing was performed as described in Materials in Methods. **(a)** Approximately 1000 time traces were averaged for both correct (red line) and mismatch (blue line) flows. **(b)** Individual time trace example for correct (red line) and mismatch (blue line) flows.

Supplementary Table 1: Common cancer panel genes and respective N-mer percentages.

Gene	1-mer	2-mer	3-mer	4-mer	5-mer	6-mer	7-mer	8-mer	9-mer	10-mer
AKT1	72.34%	22.29%	3.24%	1.02%	0.56%	0.00%	0.00%	0.00%	0.00%	0.00%
BRAF	71.84%	20.39%	4.92%	1.19%	0.77%	0.00%	0.06%	0.00%	0.00%	0.00%
GNAQ	76.48%	18.09%	3.74%	1.45%	0.12%	0.00%	0.00%	0.00%	0.00%	0.00%
GNA11	75.00%	21.29%	2.99%	0.48%	0.00%	0.12%	0.00%	0.00%	0.00%	0.00%
KIT	72.77%	19.85%	5.38%	1.53%	0.19%	0.05%	0.00%	0.00%	0.00%	0.00%
KRAS	72.53%	19.52%	5.30%	1.69%	0.48%	0.00%	0.00%	0.00%	0.00%	0.00%
NRAS	73.47%	19.95%	4.69%	0.94%	0.23%	0.23%	0.00%	0.00%	0.00%	0.00%
PIK3CA	71.90%	19.10%	5.94%	1.51%	0.65%	0.09%	0.04%	0.00%	0.00%	0.00%
EGFR	72.63%	19.46%	5.36%	1.42%	0.49%	0.07%	0.00%	0.00%	0.00%	0.00%
FGFR2	71.29%	21.84%	4.56%	1.28%	0.45%	0.06%	0.00%	0.00%	0.00%	0.00%
MET	73.06%	18.40%	5.99%	1.57%	0.46%	0.03%	0.00%	0.00%	0.00%	0.00%
PDGFRA	73.32%	19.47%	4.94%	1.19%	0.45%	0.08%	0.00%	0.00%	0.00%	0.00%
EML4	74.10%	18.04%	5.37%	1.65%	0.28%	0.05%	0.05%	0.05%	0.00%	0.00%
ALK	70.37%	21.88%	5.04%	1.62%	0.46%	0.09%	0.00%	0.00%	0.00%	0.00%
TP53	72.72%	18.61%	4.86%	1.50%	0.81%	0.35%	0.00%	0.00%	0.00%	0.00%
PTEN	72.94%	18.15%	5.75%	1.35%	0.68%	0.23%	0.00%	0.00%	0.00%	0.00%
ERBB2	73.55%	17.97%	5.26%	1.89%	0.56%	0.11%	0.00%	0.00%	0.00%	0.00%
ERCC1	70.22%	20.14%	7.06%	2.24%	0.17%	0.00%	0.00%	0.00%	0.00%	0.00%
MAGEA3	71.11%	20.82%	5.13%	2.05%	0.44%	0.00%	0.00%	0.00%	0.00%	0.00%
PDGFRB	74.90%	18.61%	4.50%	1.12%	0.40%	0.04%	0.00%	0.00%	0.00%	0.00%
RRM1	73.76%	19.17%	5.66%	1.30%	0.06%	0.00%	0.00%	0.00%	0.00%	0.00%
VEGFA	72.09%	19.16%	5.20%	1.33%	1.00%	0.00%	0.00%	0.11%	0.00%	0.00%
VEGFR2	72.01%	20.30%	5.17%	1.44%	0.50%	0.03%	0.00%	0.00%	0.00%	0.00%
CFTR	71.36%	20.38%	5.25%	1.52%	0.47%	0.22%	0.06%	0.00%	0.00%	0.00%
HEXA	72.75%	20.80%	4.67%	1.44%	0.17%	0.00%	0.00%	0.00%	0.00%	0.00%
IKBKAP	72.80%	19.65%	5.45%	1.08%	0.37%	0.14%	0.00%	0.00%	0.00%	0.00%
ASPA	70.94%	19.08%	5.37%	2.24%	1.04%	0.00%	0.15%	0.00%	0.00%	0.00%
FANCC	73.68%	19.12%	4.80%	1.12%	0.48%	0.16%	0.00%	0.00%	0.00%	0.00%
SMPD1	72.16%	19.45%	5.64%	1.30%	0.43%	0.29%	0.00%	0.00%	0.00%	0.00%
BLM	72.63%	17.90%	5.66%	2.05%	0.62%	0.16%	0.07%	0.00%	0.03%	0.00%
MCOLN1	75.08%	19.13%	3.84%	0.90%	0.38%	0.15%	0.00%	0.00%	0.00%	0.00%
GBA	73.68%	19.11%	5.28%	1.59%	0.08%	0.08%	0.00%	0.00%	0.00%	0.00%
G6PC	73.30%	19.52%	5.42%	1.51%	0.13%	0.00%	0.00%	0.00%	0.00%	0.00%
BCKDHA	73.55%	19.84%	4.01%	1.40%	0.50%	0.00%	0.10%	0.00%	0.00%	0.00%
BCKDHB	70.71%	20.12%	4.64%	2.62%	0.71%	0.24%	0.00%	0.00%	0.00%	0.00%
DLD	71.80%	20.64%	5.52%	0.80%	0.53%	0.00%	0.09%	0.00%	0.00%	0.00%
SMN	68.56%	21.39%	6.32%	2.43%	0.49%	0.16%	0.00%	0.00%	0.00%	0.00%
ABCC8	74.12%	20.40%	3.93%	1.06%	0.20%	0.06%	0.00%	0.00%	0.00%	0.00%
NEB	73.57%	19.50%	4.89%	1.21%	0.33%	0.08%	0.01%	0.00%	0.00%	0.00%
PCDH15	72.38%	20.29%	4.74%	1.34%	0.46%	0.12%	0.05%	0.00%	0.00%	0.00%
CLRN1	71.15%	17.15%	6.24%	3.51%	0.19%	0.78%	0.00%	0.00%	0.00%	0.00%
HBB	71.78%	21.17%	6.13%	0.92%	0.00%	0.00%	0.00%	0.00%	0.00%	0.00%
HBA1	68.91%	25.96%	3.85%	1.28%	0.00%	0.00%	0.00%	0.00%	0.00%	0.00%
HBA2	68.91%	25.96%	3.85%	1.28%	0.00%	0.00%	0.00%	0.00%	0.00%	0.00%
